# Independent prognostic value of the gene-only BCT Score compared with the 21-gene recurrence score in ER+/HER2− early breast cancer

**DOI:** 10.3389/fonc.2026.1692019

**Published:** 2026-03-26

**Authors:** Sung Gwe Ahn, Jonghan Yu, Sang Uk Woo, Seung Ho Baek, Woo Young Kim, Jai Hyun Chung, Soong June Bae, Seok Won Kim, Seok Jin Nam, Gyungyub Gong, Young-Won Lee, Jai Hong Han, Joon Jeong, Eun-Gyeong Lee, Sae Byul Lee

**Affiliations:** 1 Department of Surgery, Gangnam Severance Hospital, Yonsei University College of Medicine, Seoul, Republic of Korea; 2 Institute for Breast Cancer Precision Medicine, Yonsei University College of Medicine, Seoul, Republic of Korea; 3 Breast Division, Department of Surgery, Samsung Medical Center, Sungkyunkwan University School of Medicine, Seoul, Republic of Korea; 4 Department of Breast and Endocrine Surgery, Korea University Guro Hospital, Seoul, Republic of Korea; 5 Department of Pathology, Asan Medical Center, University of Ulsan College of Medicine, Seoul, Republic of Korea; 6 Department of Surgery, Asan Medical Center, University of Ulsan College of Medicine, Seoul, Republic of Korea; 7 Department of Surgery, Research Institute and Hospital, National Cancer Center, Goyang, Republic of Korea

**Keywords:** BCT gene score, BCT score, breast cancer, ER+HER2- breast cancer, multigene assay

## Abstract

**Background:**

The GenesWell BCT is a prognostic assay that integrates the expression of six prognostic and three reference genes with clinicopathological factors (tumor size and nodal status) to generate a BCT Score. The BCT Score is used to predict the risk of recurrence in early breast cancer. We excluded clinical variables and developed a gene-only version of the BCT Gene Score to evaluate its independent prognostic performance.

**Methods:**

We assessed the prognostic value of the BCT Gene Score in a cohort of patients with ER+/HER2− early breast cancer using available Oncotype DX Recurrence Score (RS) data. The primary endpoint was concordance between the assays and recurrence-free survival (RFS).

**Results:**

We analyzed 759 samples from five Korean institutions. Risk classification concordance between the BCT Gene Score and the BCT Score was 81.2%, whereas concordance with the Oncotype DX RS was 74.2%, indicating moderate agreement. All three scores were significantly associated with RFS (*p* < 0.05). Combining the BCT and BCT Gene Scores improved prognostic stratification compared with either score alone. Patients classified as high risk by both scores exhibited the poorest prognosis, whereas those classified as low risk by both scores exhibited the most favorable outcomes. Among patients with low RS, those reclassified as high risk by the BCT Gene Score exhibited significantly worse RFS.

**Conclusions:**

The BCT Gene Score exhibits independent prognostic utility and complements the original BCT Score. Risk stratification may be enhanced by incorporating both scores, ultimately guiding more precise treatment decisions in ER+/HER2− early breast cancer.

## Introduction

Multigene assays (MGAs) have been employed to guide adjuvant treatment decisions in patients with estrogen receptor-positive (ER+)/HER2− early breast cancer (1, 2). For example, Prosigna and EndoPredict integrate molecular data with clinical parameters such as tumor size and nodal status (3, 4).

The Breast Cancer Test (BCT) score is a commercially available multigene signature in Korea developed using tumor samples from patients with ER+/HER2− breast cancer. It combines the expression of six prognostic and three reference genes with clinical factors (tumor size and nodal status) to generate a prognostic score (5). Its prognostic utility has been demonstrated in multiple independent cohorts, including studies involving Asian populations (6, 7).

A previous analysis using a cohort treated with adjuvant therapy based on the 21-gene recurrence score (RS) revealed that the BCT score provided additional prognostic information, particularly among patients with low RS (8).

In this study, we evaluated the prognostic value of the BCT Gene Score independent of clinical parameters. The BCT Score refers to the original algorithm integrating gene expression and clinicopathologic variables, whereas the BCT Gene Score represents the gene-only model constructed by excluding clinical factors from the original formula. Using the same cohort from our previous study comparing the BCT Score and RS, we analyzed the performance of the BCT Gene Score as a standalone molecular signature. The primary objective of this study was to evaluate the independent prognostic value of the gene-only BCT Score in patients with ER+/HER2− early breast cancer with available 21-gene RS data. Secondary objectives included comparison of risk classification between assays and assessment of additional prognostic stratification within the low-RS population.

## Materials and methods

### Study design and patient population

We retrospectively analyzed previously collected data from patients with ER+/HER2− early-stage breast cancer (pathologic T1–2, N0–1) who underwent curative surgery at five participating institutions. The same patient cohort was used in a previous multicenter study that compared the prognostic value of the BCT Score and RS. Details of the patient cohort have been described previously (8). Eligible patients had available RS results that were used to guide adjuvant treatment decisions according to institutional practice. Survival and recurrence information were retrospectively collected between July and December 2022, covering the period from surgery to the last follow-up or death. Formalin-fixed, paraffin-embedded tumor samples from these patients were used to ensure consistency in the molecular analysis. We specifically focused on patients for whom multigene assay (MGA) results (RS and BCT Score) were available, allowing us to evaluate the newly derived BCT Gene Score. The study was approved by the Institutional Review Board (IRB) of the participating institutions, and informed consent was waived in accordance with ethical guidelines.

### BCT Gene Score

The BCT Gene Score was developed as a modified molecular prognostic model based on the previously validated BCT Score. Clinical variables (tumor size and nodal status) were removed from the original algorithm to construct a gene-only model, allowing evaluation of the intrinsic prognostic contribution of gene expression independent of clinicopathologic factors.

The gene composition was identical to that of the validated BCT Score, which was originally developed based on biological relevance and statistical association with distant metastasis in HR+/HER2− early breast cancer. No re-selection of genes or re-estimation of gene coefficients was performed in this study.

The original BCT Score was calculated as follows (5):


$$\begin{array}{c} Unscaled\;BCT\;Score=\left(0.63\times \text{Δ}Ct_{UBE2C}\right)+\left(0.32\times \text{Δ}Ct_{TOP2A}\right)+\left(0.13\times \text{Δ}Ct_{RRM2}\right)\\ +\left(0.02\times \text{Δ}Ct_{FOXM1}\right)+\left(0.04\times \text{Δ}Ct_{MKI67}\right)-\left(0.42\times \text{Δ}Ct_{BTN3A2}\right)\\ +\left(0.89\times Tumor\;size\right)+\left(1.22\times pN\;status\right) \end{array}$$

BCT Score=(0.8×Unscaled BCT Score)−13.71

Tumor size and nodal status were removed from the equation to derive the BCT Gene Score, and anadjustment constant (C_adj._) was added to preserve the original risk group distribution. The (C_adj._) was optimized to ensure consistency in the proportion of low- and high-risk patients (using a cutoff score of 4) with the original BCT Score reference cohort (*n* = 759), in which 68.5% of patients were classified as low risk and 31.5% as high risk. The final (C_adj._) was 2.034, yielding 68.6% low-risk and 31.4% high-risk distribution when applied to the gene-only model (**Supplementary Figure 1**).

The BCT Gene Score was thus computed as follows:


$$\begin{array}{c} Unscaled\;BCT\;Gene\;Score\\ =\left(0.63\times \text{Δ}Ct_{UBE2C}\right)+\left(0.32\times \text{Δ}Ct_{TOP2A}\right)+\left(0.13\times \text{Δ}Ct_{RRM2}\right)\\ +\left(0.02\times \Delta Ct_{FOXM1}\right)+\left(0.04\times \Delta Ct_{MKI67}\right)-\left(0.42\times \Delta Ct_{BTN3A2}\right)+C_{adj.} \end{array}$$

BCT Gene Score=(0.8×Unscaled BCT Gene Score)−13.71

The BCT Gene Score was normalized to a 0–10 scale. Values below 0 were set to 0, and values exceeding 10 were capped at 10. Patients were stratified into low-risk (<4) and high-risk (≥4) groups based on a predefined cutoff of 4.

### Concordance analysis

Subsequently, we evaluated the agreement among the risk classifications based on the RS, BCT Score, and BCT Gene Score. The TAILORx threshold was used for the RS, with scores ≤25 classified as low risk and scores >25 as high risk (9). The validated cutoff of 4 was used for both the BCT Score and BCT Gene Score, with scores<4 and ≥4 considered low risk and high risk, respectively (5). Concordance was expressed as the overall concordance rate, defined as the proportion of patients classified into the same risk category by both assays. In addition, we conducted sub-analyses stratified by nodal status (pN0 *vs*. pN1) to explore potential differences in concordance between groups.

### Statistical analysis

The primary objective was to evaluate whether the BCT Gene Score independently predicts recurrence-free survival (RFS) in patients with ER+/HER2− early breast cancer with available 21-gene Recurrence Score (RS) results. To address this objective, survival analyses were conducted as follows. First, the prognostic performance of each assay (BCT Gene Score, BCT Score, and RS) was assessed using Kaplan–Meier survival analysis, and differences between predefined risk groups were evaluated using the log-rank test. Second, multivariate Cox proportional hazards regression models were constructed to determine whether the BCT Gene Score retained independent prognostic significance after adjustment for age, tumor size, nodal status, and histologic grade. Each score was analyzed as both as a categorical variable (risk group) and as a continuous variable. Third, subgroup analyses were performed among patients classified as low risk by the RS to evaluate whether the BCT Gene Score provided additional prognostic stratification beyond RS-based assessment.

Baseline characteristics between the groups were compared by analyzing categorical variables using the chi-square test or Fisher’s exact test, as appropriate. All statistical analyses were performed using R software (version 4.3.1), with a two-sided *p*-value<0.05 considered significant.

## Results

### Patient characteristics

This cohort represents the same patient population previously reported in our multicenter study evaluating the prognostic value of the BCT Score and the 21-gene Recurrence Score. Detailed information regarding adjuvant treatment patterns and overall clinicopathologic characteristics has been described previously (8). In brief, most enrolled patients were women aged ≤50 years with small (≤2 cm), node-negative, ER+/HER2− tumors (**Table 1**). The median age at surgery was 49 years (range: 27–79), and 514 patients (66.7%) were ≤50 years of age. Tumor size was ≤2 cm in 504 patients (65.4%), and only 151 patients (19.9%) had one to three metastatic axillary lymph nodes.

**Table 1:** Clinicopathological characteristics of the study cohort according to the BCT Gene Score, BCT Score, and Recurrence Score.

	All	BCT score			BCT Gene score			Recurrence score		
		Low	High	P-value	Low	High	P-value	Low	High	P-value
*n*	759	520	239	–	521	238	–	645	114	–
Age				0.806			0.602			0.172
*≤50*	505 (66.5%)	344 (66.2%)	161 (67.4%)		343 (65.8%)	162 (68.1%)		436 (67.6%)	69 (60.5%)	
*>50*	254 (33.5%)	176 (33.8%)	78 (32.6%)		178 (34.2%)	76 (31.9%)		209 (32.4%)	45 (39.5%)	
Size				**<0.001**			**0.004**			**0.002**
*≤2 cm*	495 (65.2%)	408 (78.5%)	87 (36.4%)		358 (86.7%)	137 (57.6%)		436 (67.6%)	59 (51.8%)	
*>2 cm*	264 (34.8%)	112 (21.5%)	152 (63.6%)		163 (31.3%)	101 (42.4%)		209 (32.4%)	55 (48.2%)	
pN				**<0.001**			**0.020**			0.287
*0*	608 (80.1%)	450 (86.5%)	158 (66.1%)		405 (77.7%)	203 (85.3%)		512 (79.4%)	96 (84.2%)	
*1*	151 (19.9%)	70 (13.5%)	81 (33.9%)		116 (22.3%)	35 (14.7%)		133 (20.6%)	18 (15.8%)	
Histologic Grade				**<0.001**			**<0.001**			**<0.001**
*1*	136 (17.9%)	119 (22.9%)	17 (7.1%)		117 (22.5%)	19 (8.0%)		129 (20.0%)	7 (6.1%)	
*2*	532 (70.1%)	358 (68.8%)	174 (72.8%)		373 (71.6%)	159 (66.8%)		469 (72.7%)	63 (55.3%)	
*3*	91 (12.0%)	43 (8.3%)	48 (20.1%)		31 (6.0%)	60 (25.2%)		47 (7.3%)	44 (38.6%)	
Nuclear Grade				**<0.001**			**<0.001**			**<0.001**
*1*	63 (8.3%)	55 (10.6%)	8 (3.3%)		55 (10.6%)	8 (3.4%)		60 (9.3%)	3 (2.6%)	
*2*	578 (76.2%)	399 (76.7%)	179 (74.9%)		419 (80.4%)	159 (66.8%)		514 (79.7%)	64 (56.1%)	
*3*	118 (15.5%)	66 (12.7%)	52 (21.8%)		47 (9.0%)	71 (29.8%)		71 (11.0%)	47 (41.2%)	
PR				0.504			**0.001**			**<0.001**
*Negative*	76 (10.0%)	49 (9.4%)	27 (11.3%)		39 (7.5%)	37 (15.5%)		44 (6.8%)	34 (28.1%)	
*Positive*	683 (90.0%)	471 (90.6%)	212 (88.7%)		482 (92.5%)	201 (84.5%)		601 (93.2%)	82 (71.9%)	
Type				0.204			**0.027**			**<0.001**
*Ductal*	636 (83.8%)	443 (85.2%)	193 (80.8%)		424 (81.4%)	212 (89.1%)		535 (82.9%)	101 (88.6%)	
*Lobular*	65 (8.6%)	43 (8.3%)	22 (9.2%)		52 (10.0%)	13 (5.5%)		62 (9.6%)	3 (2.6%)	
*Others*	58 (7.6%)	34 (6.5%)	24 (10.0%)		45 (8.6%)	13 (5.5%)		48 (7.4%)	10 (8.8%)	

Bold values mean a statistical significance.

Patients were classified based on risk stratification by MGA as follows: 239 patients (31.5%), 238 patients (31.3%), and 114 patients (15.0%) were high risk according to the BCT Score, BCT Gene Score, and RS, respectively. Across all three signatures—BCT Gene Score, BCT Score, and RS—the high-risk groups were significantly associated with larger tumor size and higher prevalence of histologic grade 3 tumors (*p* < 0.050) (**Table 1**).

Significant differences in anatomical tumor burden were observed among the prognostic signatures despite the presence of common characteristics. Although all three prognostic signatures exhibited a higher proportion of tumors >2 cm in the high-risk group (*p* < 0.050), the highest enrichment was observed for the BCT Score (*n* = 152, 63.6%), which incorporated tumor size and nodal status into the prognostic algorithm. In addition, the BCT Score identified a significantly higher proportion of high-risk patients among those with pN1 tumors than among those with pN0 tumors (*p* < 0.001). In contrast, the proportion of pN1 patients was not significantly higher in the high-risk group than in the low-risk group for either the RS or the BCT Gene Score.

Differences in risk classification were observed with respect to the progesterone receptor (PR) status and cancer type. The proportion of PR-negative tumors was significantly higher in the high-risk group than in the low-risk group (*p* < 0.001) according to the RS, which includes the expression of estrogen-related genes such as *ESR1*, *PGR*, *BCL2*, and *SCUBE2* (10). Although the BCT Gene Score does not include estrogen-related genes, a high BCT Gene Score was associated with negative PR (*p* = 0.001). Additionally, lobular carcinoma was more frequently observed in the low-risk groups classified by both the BCT Gene Score and RS (*p* < 0.050).

### Concordance analysis

The overall concordance rate between the BCT Gene Score and the BCT Score (BS) for risk classification was 81.2% (616/759) (**Table 2**). However, concordance varied according to the nodal status. The concordance between the BCT Score and BCT Gene Score was 84.0% (511/608) among node-negative patients. Within this group, 15.8% (71/450) of low-risk patients were reclassified as high risk, whereas 16.5% (26/158) of high-risk patients were reclassified as low risk based on the BCT Gene Score. The concordance rate among node-positive patients was lower, at 69.5% (105/151). Notably, all patients classified as low risk by the BCT Score remained low risk according to the BCT Gene Score, whereas 56.8% (46 out of 81) of high-risk patients were reclassified as low risk according to the BCT Gene Score.

**Table 2:** Concordance rates for risk classification among the BCT Gene Score, BCT Score, and Recurrence Score.

		All (n = 759)			Lymph node negative (n = 608)			Lymph node positive (n = 151)		
		BCT score			BCT score			BCT score		
		Low (BS<4)	High (BS ≥4)	Total	Low (BS<4)	High (BS ≥4)	Total	Low (BS<4)	High (BS ≥4)	Total
BCT Gene Score	Low (BGS<4)	449 (59.2%)	72 (9.5%)	521 (68.6%)	379 (62.3%)	26 (4.3%)	405 (66.6%)	70 (46.4%)	46 (30.5%)	116 (76.8%)
	High (BGS ≥4)	71 (9.4%)	167 (22.0%)	238 (31.4%)	71 (11.7%)	132 (21.7%)	203 (33.4%)	0 (0.0%)	35 (23.2%)	35 (23.2%)
	Total	520 (68.5%)	239 (31.5%)	759 (100.0%)	450 (74.0%)	158 (26.0%)	608 (100.0%)	70 (46.4%)	81 (53.6%)	151 (100.0%)
		Recurrence Score			Recurrence Score			Recurrence Score		
		Low (RS 0–25)	High (RS ≥26)	Total	Low (RS 0–25)	High (RS ≥26)	Total	Low (RS 0–25)	High (RS ≥26)	Total
BCT Score	Low (BS<4)	476 (62.7%)	44 (5.8%)	520 (68.5%)	410 (67.4%)	40 (6.6%)	450 (74.0%)	66 (43.7%)	4 (2.6%)	70 (46.4%)
	High (BS ≥4)	169 (22.3%)	70 (9.2%)	239 (31.5%)	102 (16.8%)	56 (9.2%)	158 (26.0%)	67 (44.5%)	14 (9.3%)	81 (53.6%)
	Total	645 (85.0%)	114 (15.0%)	759 (100.0%)	512 (84.2%)	96 (15.8%)	608 (100.0%)	133 (88.1%)	18 (11.9%)	151 (100.0%)

Pairwise concordance in risk classification between the BCT Gene Score, BCT Score, and Recurrence Score (RS) in the overall cohort and stratified by nodal status. Risk groups were defined using a cut-off score of 4 for the BCT Gene Score and BCT Score, and the TAILORx threshold (RS ≤25 as low risk and RS >25 as high risk) for the Recurrence Score.

The overall concordance rate between the BCT Gene Score and RS was 74.2% (563/759) (**Table 2**), comparable to the previously reported concordance rate of 71.9% between these two scores (8). Unlike the comparison with the BCT Score, no significant difference in concordance according to nodal status was observed between the two risk classification methods. The concordance rate was 73.6% (447/608) among node-negative patients and 76.7% (116/151) among node-positive patients.

### Survival analysis according to the BCT Gene Score

**Figure 1:**
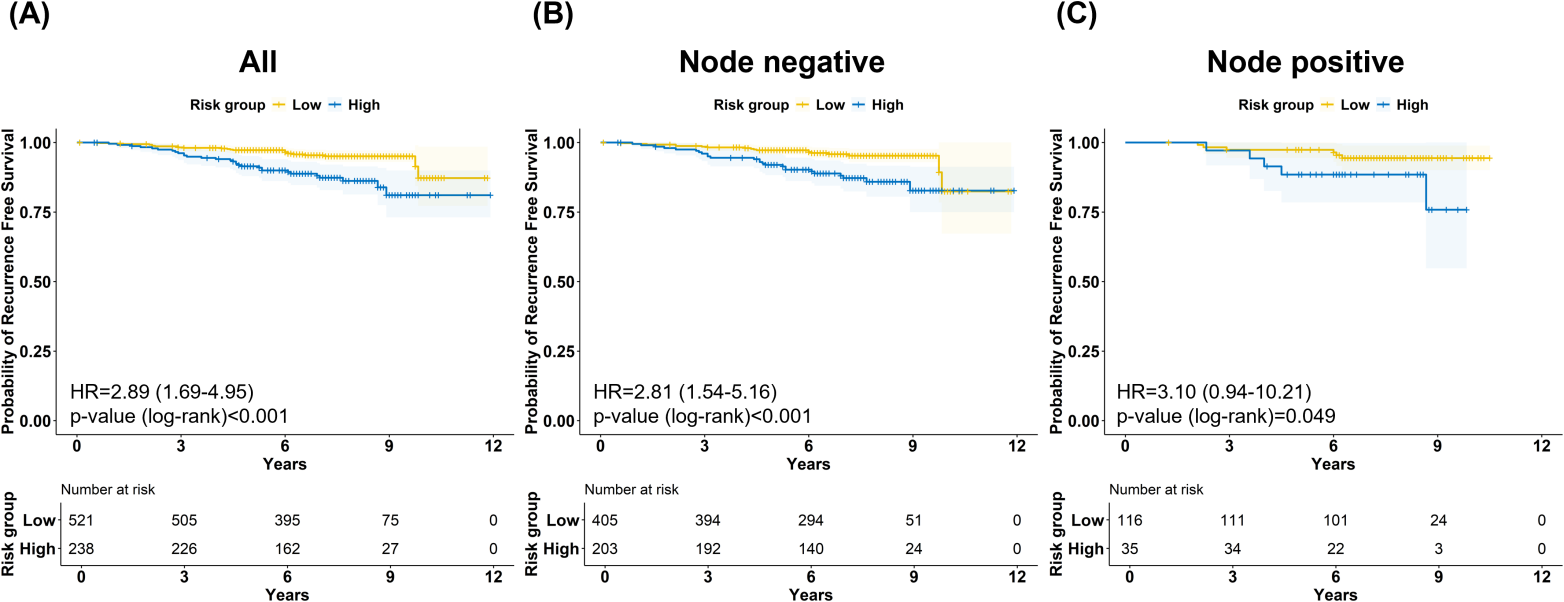
Recurrence-free survival according to BCT Gene Score. Kaplan–Meier curves of recurrence-free survival (RFS) stratified by BCT Gene Score in **(A)** the overall cohort, **(B)** lymph node-negative patients, and **(C)** lymph node-positive patients. The high-risk group demonstrated significantly poorer RFS compared with the low-risk group in the overall cohort (p<0.001), in node-negative patients (p<0.001), and in node-positive patients (p = 0.049).

RFS was evaluated using the BCT Gene Score. At a median follow-up of 7 years, the 7-year RFS rates in the low-risk and high-risk groups were 95.4% (93.5%–97.4%) and 87.3% (82.9%–92.0%), respectively, with an HR of 2.89 (1.69–4.95; *p* < 0.001) (**Figure 1A**).

Among node-negative patients, the 7-year RFS rates were 95.8% (93.7%–98.0%) and 87.2% (82.4%–92.4%) in the low-risk and high-risk groups, respectively, corresponding to an HR of 2.81 (1.54–5.16) (**Figure 1B**). Among node-positive patients, the 7-year RFS rates were 94.4% (90.1%–98.9%) and 88.5% (78.5%–99.8%) in the low-risk and high-risk groups, respectively, with an HR of 3.10 (0.94–10.21) (**Figure 1C**).

Survival outcomes were assessed according to the BCT score and RS (**Supplementary Figure 2**). Although both scores were significant in node-negative patients (*p<*0.050), only the BCT Score maintained significant prognostic value for node-positive disease.

The BCT Gene Score was further validated as an independent prognostic factor using multivariate Cox proportional hazards models adjusted for age, tumor size, histologic grade, and nodal status (**Table 3**). When analyzed as a categorical variable (low *vs*. high risk), the BCT Gene Score was significantly associated with recurrence risk (adjusted HR = 2.56 [1.45–4.52]). A similar association was observed when the BCT Gene Score was analyzed as a continuous variable (adjusted HR = 1.95 [1.41–2.71]), indicating that higher scores were associated with increased recurrence risk.

**Table 3:** Multivariate Cox proportional hazards analysis for recurrence-free survival according to the BCT Gene Score, BCT Score, and Recurrence Score.

BCT Gene Score					
Variable	HR (95% C.I.)	p-value	Variable	HR (95% C.I.)	p-value
BCT gene risk group (Low *vs*. High)	2.56 (1.45–4.52)	**0.001**	BCT gene score (z-score scaled)	1.95 (1.41–2.71)	**<0.001**
Age (≤50 *vs*. >50)	0.67 (0.36–1.26)	0.218	Age (≤50 *vs*. >50)	0.68 (0.36–1.28)	0.231
Size (≤2 *vs*. >2)	1.13 (0.65–1.96)	0.674	Size (≤2 *vs*. >2)	1.09 (0.62–1.90)	0.763
Grade (1 and 2 *vs*. 3)	1.69 (0.88–3.26)	0.113	Grade (1 and 2 *vs*. 3)	1.48 (0.76–2.87)	0.247
pN (0 *vs*. 1)	1.12 (0.57–2.18)	0.742	pN (0 *vs*. 1)	1.16 (0.59–2.26)	0.668
(B) BCT Score					
BCT Score					
Variable	HR (95% C.I.)	p-value	Variable	HR (95% C.I.)	p-value
BCT risk group (Low *vs*. High)	2.61 (1.40–4.84)	**0.002**	BCT score (z-score scaled)	2.21 (1.56–3.12)	**<0.001**
Age (≤50 *vs*. >50)	0.69 (0.37–1.30)	0.251	Age (≤50 *vs*. >50)	0.69 (0.37–1.29)	0.245
Size (≤2 *vs*. >2)	0.81 (0.44–1.50)	0.506	Size (≤2 *vs*. >2)	0.64 (0.34–1.21)	0.167
Grade (1 and 2 *vs*. 3)	1.94 (1.02–3.68)	**0.043**	Grade (1 and2 *vs*. 3)	1.47 (0.77–2.84)	0.245
pN (0 *vs*. 1)	0.77 (0.39–1.53)	0.457	pN (0 *vs*. 1)	0.66 (0.33–1.32)	0.242
(C) Recurrence Score					
Recurrence Score					
Variable	HR (95% C.I.)	p-value	Variable	HR (95% C.I.)	p-value
RS risk group (0–25 *vs*. 26–100)	1.64 (0.82–3.27)	0.161	Recurrence score (z-score scaled)	1.30 (1.02–1.67)	**0.036**
Age (≤50 *vs*. >50)	0.65 (0.35–1.22)	0.178	Age (≤50 *vs*. >50)	0.66 (0.35–1.23)	0.192
Size (≤2 *vs*. >2)	1.18 (0.68–2.06)	0.562	Size (≤2 *vs*. >2)	1.21 (0.69–2.09)	0.506
Grade (1 and 2 *vs*. 3)	1.89 (0.93–3.82)	0.077	Grade (1 and 2 *vs*. 3)	1.57 (0.74–3.35)	0.238
pN (0 *vs*. 1)	1.00 (0.52–1.95)	0.992	pN (0 *vs*. 1)	1.04 (0.53–2.02)	0.919

Hazard ratios (HR) with 95% confidence intervals (CI) for recurrence-free survival are shown for each assay when analyzed as a categorical variable (left) and as a continuous variable (right). Bold values mean a statistical significance.

For the other two signatures, the BCT Score retained independent prognostic significance, both as a categorical variable (adjusted HR = 2.61 [1.40–4.84]) and as a continuous variable (adjusted HR = 2.21 [1.56–3.12]). RS as a continuous variable (adjusted HR = 1.30 [1.02–1.67]) was significantly associated with recurrence; however, as a categorical variable, it was not significantly associated (adjusted HR = 1.64 [0.82–3.27]).

### Prognostic value of the combined model with BCT Gene Score and BCT Score

**Figure 2:**
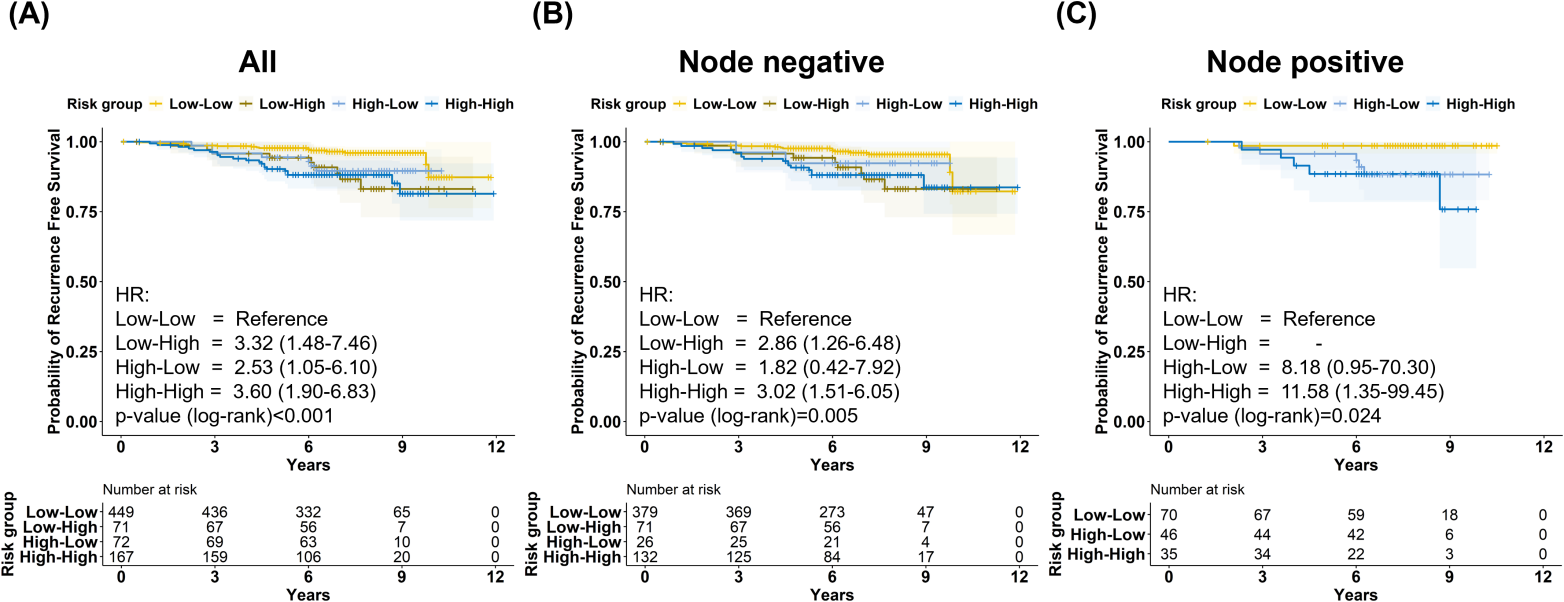
Recurrence-free survival according to risk classification between BCT Score and BCT Gene Score. Kaplan–Meier curves of recurrence-free survival (RFS) stratified by combined BCT Score and BCT Gene Score risk groups in **(A)** the overall cohort, **(B)** lymph node-negative patients, and **(C)** lymph node-positive patients. Patients were categorized into four groups: BCT Score-low/BCT Gene Score-low (Low–Low), BCT Score-low/BCT Gene Score-high (Low–High), BCT Score-high/BCT Gene Score-low (High–Low), and BCT Score-high/BCT Gene Score-high (High–High).

Patients were stratified into four groups based on the BCT Score and BCT Gene Score. RFS was highest in the double low-risk group and lowest in the double high-risk group (**Figure 2A**). This trend remained consistent in node-negative patients (**Figure 2B**); however, it was not statistically significant in the node-positive subgroup (**Figure 2C**).

Among the discordant groups, patients classified as BCT Score-low/BCT Gene Score-high had poorer RFS than those in the double low-risk group in both the overall and node-negative populations (**Supplementary Table 1**). The BCT Score-low/BCT Gene Score-high group in node-positive patients showed a trend toward better RFS than the double-low-risk group.

### Prognostic value of BCT Gene Score in the group with low RS

**Figure 3:**
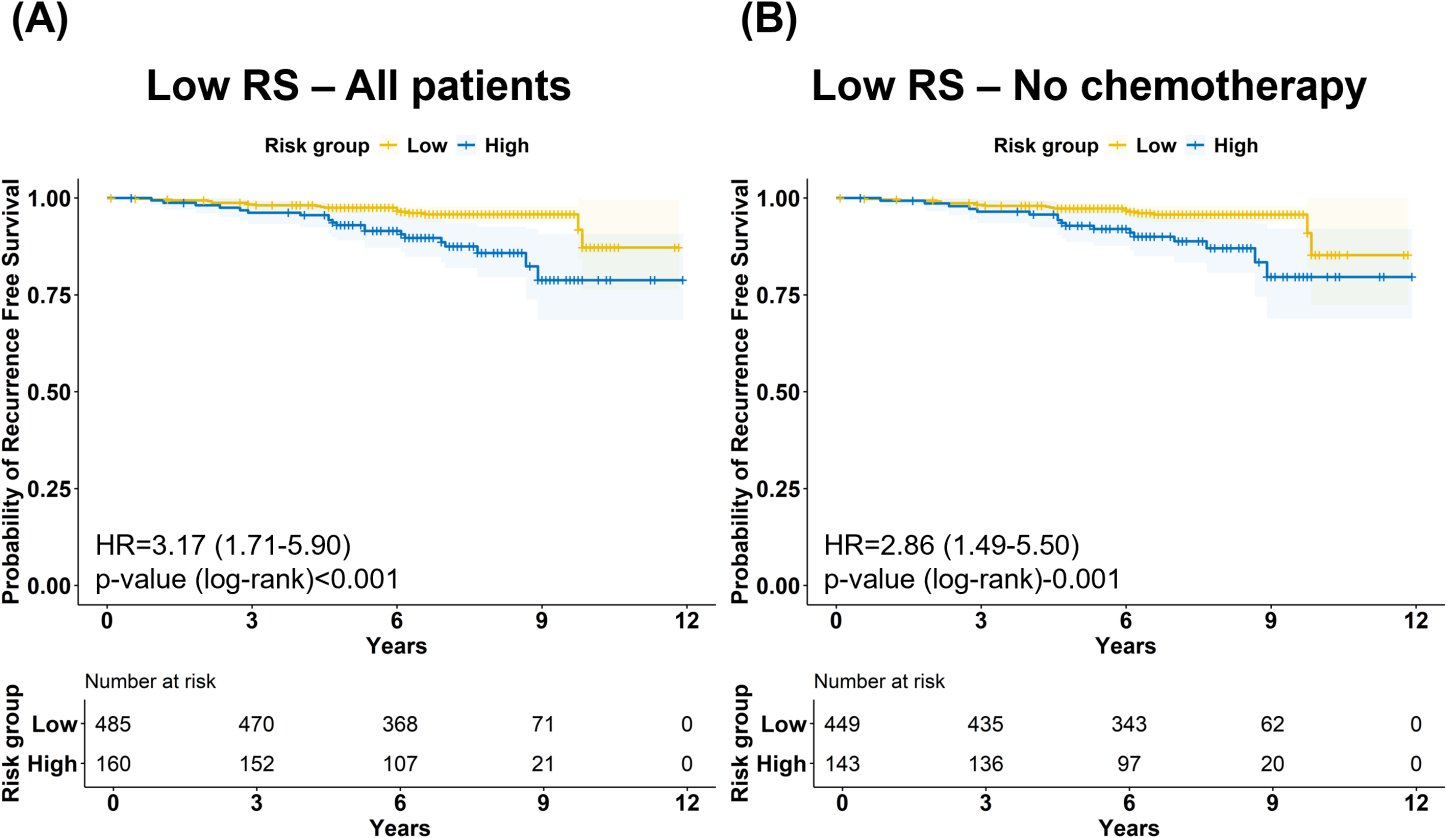
Recurrence-free survival according to BCT Gene Score in patients with low Recurrence Score. Kaplan–Meier curves show recurrence-free survival (RFS) stratified by BCT Gene Score in patients with low Recurrence Score (RS) in **(A)** the overall low-RS group and **(B)** the low-RS group without adjuvant chemotherapy. In both analyses, the high-risk group defined by the BCT Gene Score showed significantly poorer RFS compared with the low-risk group (p<0.050).

We further evaluated the prognostic utility of the BCT Gene Score in patients with low RS (*n* = 645). RFS significantly differed by BCT Gene Score within this group, with poorer outcomes observed in the high-risk BCT Gene Score subgroup (HR = 3.17 [1.71–5.90]) (**Figure 3A**). This prognostic distinction remained consistent among the 592 patients with low RS who did not receive chemotherapy, with the high-risk group showing poorer RFS (HR = 2.86 [1.49–5.50]) (**Figure 3B**).

## Discussion

Although integrating clinicopathologic factors into the BCT Score improves prognostic performance, it remains unclear whether the molecular component alone can effectively stratify risk in patients with ER+/HER2− early breast cancer. We demonstrate that the BCT Gene Score retains the prognostic value as a standalone molecular signature, independent of clinicopathological information. This finding suggests that it captures intrinsic tumor biology associated with adverse outcomes in ER+/HER2− early breast cancer.

The BCT Gene Score was derived from nine genes: six prognostic genes (*UBE2C*, *TOP2A*, *RRM2*, *FOXM1*, *MKI67*, and *BTN3A2*) and three reference genes (5). All prognostic genes, except *BTN3A2*, are associated with proliferation and overlap with components of other assays, such as PAM50. Proliferation markers are highly relevant in the luminal HER2− subtype. In contrast to the other proliferation-associated genes, *BTN3A2* is involved in immune regulation and has been implicated in immune infiltration in triple-negative breast cancer (11). We previously demonstrated a link between *BTN3A2* expression and improved distant metastasis-free survival, potentially through modulation of T cell activity (12). These findings suggest that *BTN3A2* has an immunologic role, and its prognostic relevance in ER+/HER2− breast cancer warrants further investigation in independent cohorts.

Notably, unlike some other MGAs, the BCT Gene Score does not include estrogen response elements, highlighting its unique gene composition. This also suggests that it is less influenced by systemic estrogen levels, which are largely determined by menopausal status.

We demonstrated that a combination of the BCT and BCT Gene Scores enhances risk stratification, thereby identifying patients with the most favorable or poor prognosis. This combined scoring approach may support more refined, individualized treatment decisions in patients with ER+/HER2− breast cancer. For patients within the “gray zone”—those with BCT Scores near the clinical cut-off—the BCT Gene Score may provide additional genomic insights to improve risk assessment and guide therapeutic planning. This approach allows clinicians to consider molecular and clinicopathologic contributions to recurrence risk separately, providing a more comprehensive understanding of an individual patient’s prognostic profile.

An additional practical advantage of the BCT Gene Score is that it can be derived from the same molecular assay used for the BCT Score, without the need for additional laboratory testing. By isolating the molecular component from clinicopathologic variables, the gene-only model allows clinicians to examine the intrinsic genomic prognostic signal, independent of tumor size and nodal status. This approach may be particularly informative in cases where a clearer understanding of the molecular contribution to recurrence risk is needed.

The TransATAC study reported that the Breast Cancer Index (BCI), Risk of Recurrence (ROR), and EPclin demonstrated prognostic value for late distant recurrence (13). Unlike BCI, ROR and EPclin incorporate clinical variables. These findings underscore the importance of integrating clinical information when assessing the risk of late recurrence and considering extended endocrine therapy.

Furthermore, substantial discordance was reported by the OPTIMA-prelim study among MGAs applied to the same tumor samples, with only approximately 39% of tumors consistently classified as low/intermediate versus high risk across all platforms (14). Similarly, Bartlett et al. emphasized that “no test is more equal than the others,” highlighting that, despite concordance at the cohort level, individual-level mismatches are frequent and clinically meaningful in these assays.

Similarly, we observed discordance between the RS and the BCT Gene Score, which was consistent with the discordance we previously reported between RS and the BCT Score. The OPTIMA-prelim study did not report such discrepancies. Furthermore, we demonstrated that the BCT Gene Score retained prognostic significance even among patients with a low RS, reinforcing its potential utility in refining risk assessment and guiding treatment decisions.

A limitation of our study is that patients were treated according to RS-guided decisions before the results of major randomized trials, such as TAILORx, were incorporated into clinical practice. Consequently, treatment decisions were not aligned with menopausal status or clinical risk, particularly for patients with intermediate RS (11–25). Furthermore, longer follow-up is warranted because of the risk of late recurrence in patients with ER+ disease.

In conclusion, the BCT Gene Score provides independent prognostic information in ER+/HER2− early breast cancer. Combined use with the BCT Score can enhance risk stratification and support personalized treatment planning.

## Data Availability

The original contributions presented in the study are included in the article/**Supplementary Material**. Further inquiries can be directed to the corresponding authors.

## Supplementary material

Supplementary Figure 1 Distribution of risk scores according to BCT Score and BCT Gene Score. Risk score distribution based on (A) the original BCT Score and (B) the BCT Gene Score. Patients were classified as low risk (blue) or high risk (red) using the cut-off score of 4.

Supplementary Figure 2 Recurrence-free survival according to BCT Score and Recurrence Score. Kaplan–Meier curves of recurrence-free survival (RFS) stratified by **(A–C)** BCT Score and **(D–F)** Recurrence Score (RS) in the overall cohort **(A, D)**, lymph node-negative patients **(B, E)**, and lymph node-positive patients **(C, F)**. Patients were classified into low- and high-risk groups using the cut-off for each assay.
