# Supplementary figures and images for: Independent prognostic value of the gene-only BCT Score compared with the 21-gene recurrence score in ER+/HER2− early breast cancer
Source: Front Oncol. 2026 Mar 26;16:1692019. doi: 10.3389/fonc.2026.1692019 (PMC13061679; doi:10.3389/fonc.2026.1692019)

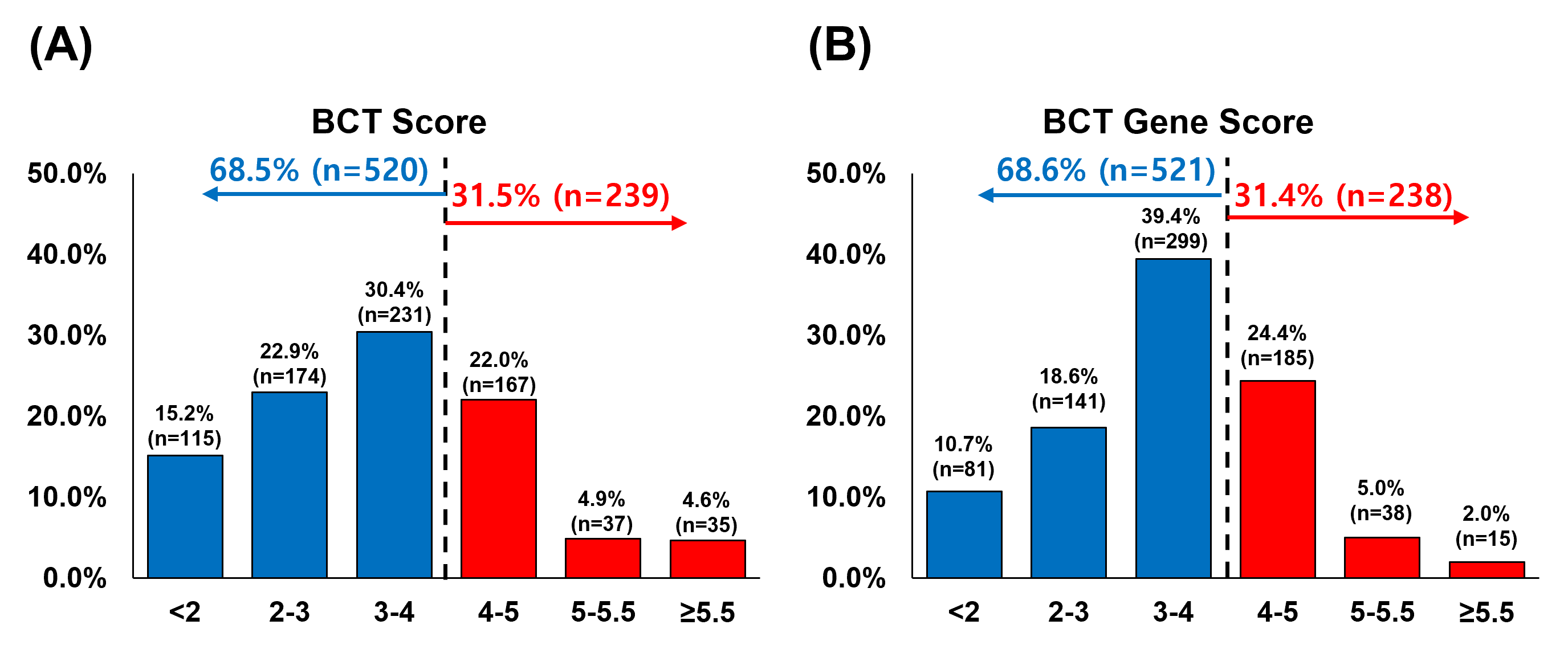

Supplement: Supplementary Figure 1 — Distribution of risk scores according to BCT Score and BCT Gene Score. Risk score distribution based on (A) the original BCT Score and (B) the BCT Gene Score. Patients were classified as low risk (blue) or high risk (red) using the cut-off score of 4. [file Image1.tif]

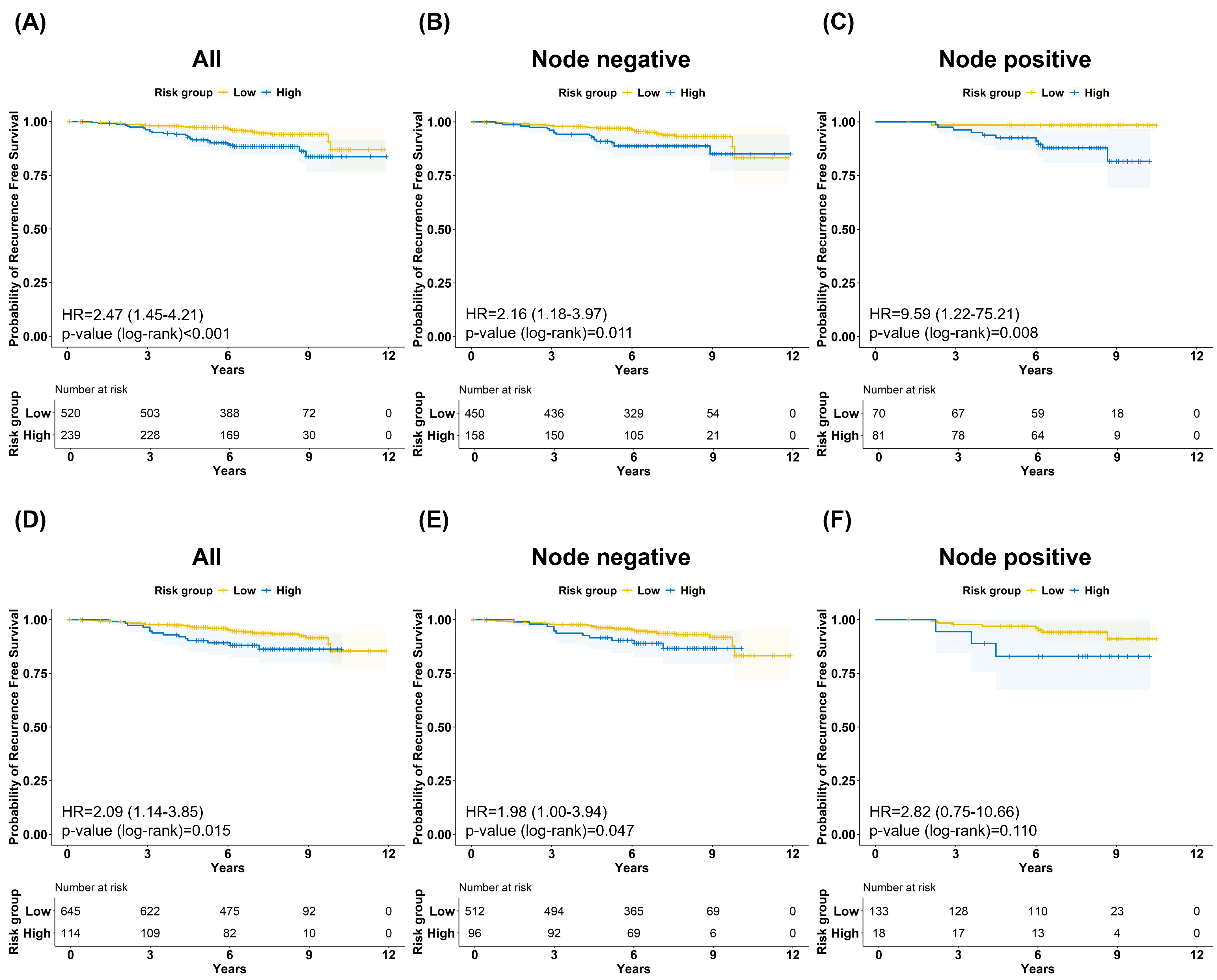

Supplement: Supplementary Figure 2 — Recurrence-free survival according to BCT Score and Recurrence Score. Kaplan–Meier curves of recurrence-free survival (RFS) stratified by (A–C) BCT Score and (D–F) Recurrence Score (RS) in the overall cohort (A, D), lymph node-negative patients (B, E), and lymph node-positive patients (C, F). Patients were classified into low- and high-risk groups using the cut-off for each assay. [file Image2.tif]
